# Reproducibility and age-related changes of ocular parametric measurements in rabbits

**DOI:** 10.1186/1746-6148-8-138

**Published:** 2012-08-19

**Authors:** Andri K Riau, Neil YS Tan, Romesh I Angunawela, Hla M Htoon, Shyam S Chaurasia, Jodhbir S Mehta

**Affiliations:** 1 Tissue Engineering and Stem Cell Group, Singapore Eye Research Institute, Singapore, Singapore; 2 Singapore National Eye Centre, Singapore, Singapore; 3 Department of Ophthalmology, Yong Loo Lin School of Medicine, National University of Singapore, Singapore, Singapore; 4 Department of Clinical Sciences, Duke-NUS Graduate Medical School, Singapore, Singapore

**Keywords:** Cornea, Rabbit, Refractive, Keratometry, Anterior chamber, Reproducibility

## Abstract

**Background:**

The rabbit is a common animal model for ophthalmic research, especially corneal research. Ocular structures grow rapidly during the early stages of life. It is unclear when the rabbit cornea becomes mature and stabilized. We investigated the changes of keratometry, refractive state and central corneal thickness (CCT) with age. In addition, we studied the intra- and inter-observer reproducibility of anterior chamber depth (ACD) and anterior chamber width (ACW) measurements in rabbits using anterior segment-optical coherence tomography (AS-OCT).

**Results:**

The growth of New Zealand White rabbits (n = 16) were monitored from age 1 to 12 months old. Corneal keratometric and refractive values were obtained using an autorefractor/keratometer, and CCT was measured using an AS-OCT. Keratometry and CCT changed rapidly from 1 to 7 months and appeared to be stabilizing after 8 months. The reduction of corneal curvature was approximately 1.36 diopter (D)/month from age 1 to 7 months, but the change decelerated to 0.30 D/month from age 8 to 12 months. An increase of 10 μm/month in CCT was observed from age 1 to 7 months, but the gain was reduced to less than 1 μm/month from age 8 to 12 months. There was a hyperopic shift over the span of 12 months, albeit the increase in spherical equivalent was slow and gradual. Rabbits of random age were then selected for 2 repeated ACD and ACW measurements by 2 independent and masked observers. Bland-Altman plots revealed a good agreement of ACD and ACW measurements inter- and intra-observer and the ranges of 95% limit of agreement were acceptable from a clinical perspective.

**Conclusions:**

Corneal keratometry, spherical equivalent refraction and CCT changed significantly during the first few months of life of rabbits. Young rabbits have been used in a large number of eye research studies. In certain settings, the ocular parametric changes are an important aspect to note as they may alter the findings made in a rabbit experimental model. In this study, we have also demonstrated for the first time a good between observer reproducibility of measurements of ocular parameters in an animal model by using an AS-OCT.

## Background

The rabbit has been a ubiquitous animal model for ocular research for decades. In the field of refractive surgery research in particular, the rabbit has been the ‘go to’ animal for evaluation of new techniques and drugs, as well as to better understand corneal wound healing responses. Refractive surgery has been rapidly evolving over last six decades [1,2]. Among the available refractive surgeries, laser in-situ keratomileusis (LASIK) is the most regularly performed procedure for the correction of myopia and hyperopia [3,4]. New techniques and technologies for refractive correction are continually emerging and hence experimental animal models have regularly been used to simulate and investigate these advances before patient application. Refractive results have often been reported from these animal models and have formed the basis for progression to human treatment.

For experimental animal models of the cornea, it is crucial that the animals share similar histological feature of the human cornea, which consists of multilayered epithelium (with thickness of approximately 50 μm), Bowman’s membrane (10 μm), stroma (450 to 500 μm), Descemet’s membrane (8 to 10 μm), and a single layer of endothelium (8 to 12 μm). With respect to these characteristics, monkey corneas are the most similar to those observed in human. However, due to economical and logistical issues, it is difficult to study refractive procedures using only monkey eyes. Other animals with corneal structure similar to human are the chicken and mouse. Studies of haze formation following femtosecond laser assisted refractive surgery have previously been reported using mouse and chicken experimental models [5,6].

However, the small size of chicken and mouse eyes makes the surgery significantly more difficult to perform. In contrast, rabbit corneas have a large diameter of approximately 15 mm [7]. The corneal epithelium, stromal lamellae arrangement, and endothelium also share similar feature as human corneas [8]. Although there are some clusters of fibrils, distinct from the rest of the anterior stroma, rabbit corneas are not generally considered to have a well developed Bowman’s membrane. Similarly, the Descemet’s membrane is present, but the collagen arrangement is found to be different than in the human corneas, which may reflect the difference in the biomechanical requirements [8]. Together with the ease of animal handling during surgery or post-operative examinations have resulted in the rabbit becoming a popular animal model to study the sequelae of various refractive procedures, such as the cataract surgery [9], LASIK [10], photorefractive keratectomy (PRK) [10,11], and refractive lenticule extraction (ReLEx) [12].

In a recent study on the feasibility of a novel technique to reverse a laser refractive surgery, we observed a significant reduction in mean corneal keratometry of our rabbit model over the 56 day-time course of the study [13]. These changes bring into question much of the published rabbit based corneal refractive research, which in many cases appear not to take these apparently growth related corneal changes into consideration. Previous studies have documented age-related changes in corneal endothelial cell count, anterior chamber depth (ACD), lens thickness, refractive power and corneal radius of curvature in rabbits [14-17]. To further define these changes in the rabbit cornea, we monitored the changes in corneal keratometry, CCT and spherical equivalent of rabbits ranging from 1 month to 12 months old. In addition, we also assessed the intra- and inter-observer variability of the measurements of ACD and anterior chamber width (ACW) in rabbits using anterior segment-optical coherence tomography (AS-OCT). Although AS-OCT was introduced almost six years ago for clinical use [18], its use for animal experimental studies is still relatively new; hence, the results of this study will be of value for researchers of corneal refractive surgery using the rabbit as an experimental model.

## Methods

### Animals

Sixteen New Zealand White rabbits (10 males and 6 females) aged 1 month-old, purchased for other research purpose, were used in this study. Measurement of ocular parameters was performed on one non-operated eye in each rabbit every month starting from age 1 month- up to 12 month-old. All rabbits were obtained from the National University of Singapore and housed under standard laboratory conditions in SingHealth Experimental Medicine Centre, Singapore. Body weight of each rabbit was measured every month before eye examination. Animals were anesthetized with xylazine hydrochloride (5 mg/kg intramuscularly; Troy Laboratories, Smithfield, Australia) and ketamine hydrochloride (50 mg/kg intramuscularly; Parnell Laboratories, Alexandria, Australia) during the measurements. The rabbits were placed near a heat source and allowed to recover from anesthesia in a quiet area. Rabbits were monitored carefully for signs of gastrointestinal stasis daily for a week. All animals were treated according to the guidelines of the Association for Research in Vision and Ophthalmology’s (ARVO) Statement for the Use of Animals in Ophthalmic and Vision Research. The study protocol was approved by the Institutional Animal Care and Use Committee of SingHealth, Singapore.

### Measurement of ocular parameters

#### Age-related changes of ocular parameters in rabbits

Measurements of CCT, corneal curvature (keratometry) and spherical equivalent refraction (refractometry) of sixteen eyes were used to represent each time point. AS-OCT scans and measurement of CCT were obtained by using a RTVue Fourier-Domain OCT (Optovue, Fremont, CA). This device has an optical resolution of 5 μm [19]. The examiner adjusted the system to position the vertex at the center of the AS-OCT image in order to maximize vertex reflection. Measurements of CCT were taken at the center (0.0 mm) and at 1 mm either side of the centre (+1.0 mm, -1.0 mm). The mean value of the three distances was then reported.

Measurements of corneal curvature and refractive error were obtained by using a Nidek ARK-30 Autorefractor/Keratometer (Hiroishi, Japan). The instrument records 10 measurements and displays a single best result that is determined automatically based on the recorded values. Keratometry and refractometry were measured for three times. The reported K-values were averages of horizontal and vertical meridian. After obtaining the spherical and cylindrical errors reading from the refractometer, spherical equivalent was then calculated using the following formula:

(1) $$\begin{array}{ll} \text{Spherical equivalent}= & \text{Spherical error}\\ & +\frac{1}{2}\text{Cylindrical error} \end{array}$$

#### Reproducibility of measurement of ocular parameters

**Figure 1:**
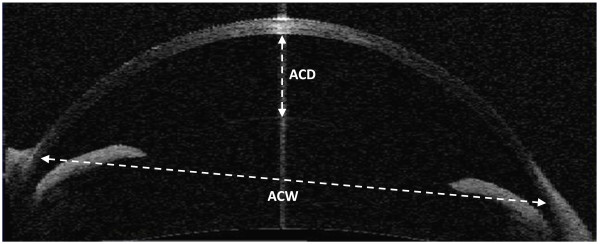
**Anterior segment OCT image of the anterior chamber in a rabbit eye.** Anterior chamber depth (ACD) was measured from the posterior surface of the center of the cornea to the apex of the crystalline lens. Anterior chamber width (ACW) was measured from the scleral spurs in the nasal and temporal quadrants of the eye.

Measurements of ACD and ACW were performed by using a Visante AS-OCT (Carl Zeiss Meditec, Jena, Germany). Data from twelve eyes of rabbits with age range of 3–10 months were analyzed for this study. The AS-OCT images of the cornea were taken along the horizontal axis (nasal-temporal angles at 0–180 degrees) using the standard anterior segment single-scan protocol. The scan was optimally aligned when the optically produced corneal reflex was visible as a vertical white line along the center of the cornea. Two trained, independent, masked observers (A. R. and N. T.) reviewed the AS-OCT scans. Using a caliper provided by the software of the Visante system, the central ACD was measured from the central posterior surface of the cornea to the apex of the crystalline lens (Figure 1). ACW was defined as the distance between the scleral spurs in the nasal and temporal quadrants (Figure 1). One week later, each observer repeated the ACD and ACW measurements on the same set of images to assess the possible intra-observer variability.

### Statistical analysis

Pearson correlation coefficient (*r* value), used to assess the correlation between-observer measurements of ACD and ACW, was analyzed using statistical software SPSS 17.0 (SPSS, Inc., Chicago, IL). The terms used to describe the strength of the correlations are as follows: very strong (|*r*| > 0.8); moderately strong (0.8 ≥ |*r*| ≥ 0.6); fairly strong (0.5 ≥ |*r*| ≥ 0.3); and poor (|*r*| < 0.3) [20]. A 2-tailed paired *t* test was used to calculate the difference between the intra- and inter-observer measurements. Bland-Altman plots were then employed to determine intra- and inter-observer agreements between the measurements [21]. MedCalc statistical software version 9.3 (MedCalc Software, Mariakerke, Belgium) was used to analyze Bland-Altman plots; 95% limits of agreement (LoA) were considered valid for Bland-Altman plots. *P*-values of less than 0.05 were considered statistically significant.

## Results

**Figure 2:**
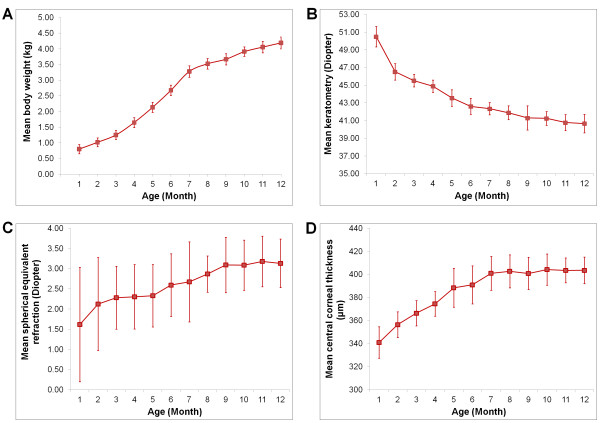
**Age-related changes in body weight (A), corneal curvature (B), refractive state (C) and central corneal thickness (D).** Error bars represent standard deviation of the mean value.

The mean values (± standard deviation) of the keratometric, spherical equivalent and CCT measurements are tabulated in Table 1. All rabbits showed a progressive weight gain. A relatively rapid weight gain was seen from month 3 to 7 with an average gain of 0.51 kg/month (Figure 2A). Similarly, keratometry and CCT also changed rapidly early on and appeared to be stabilizing after 8 months. The reduction of corneal curvature was approximately 1.36 D/month from age 1 to 7 months, but the change decelerated to 0.30 D/month from age 8 to 12 months (Figure 2B). A rate of increase of approximately 10 μm/month in CCT was observed from age 1 to 7 months, but the gain was reduced to less than 1 μm/month from age 8 to 12 months (Figure 2D). There was a hyperopic shift over the span of 12 months, albeit the increase in spherical equivalent was slow and gradual (Figure 2C).

**Table 1:** Mean keratometry, spherical equivalent and central corneal thickness of rabbits ranging from 1 month to 12 months old

**Age (months)**	Mean keratometry (D)^*^	Mean spherical equivalent (D)*	Mean CCT (μm)^**^
1	50.50 ± 1.15	1.62 ± 1.41	341 ± 14
2	46.52 ± 0.73	2.13 ± 1.15	356 ± 11
3	45.52 ± 0.71	2.28 ± 0.78	366 ± 11
4	44.89 ± 0.69	2.30 ± 0.80	374 ± 11
5	43.56 ± 0.93	2.33 ± 0.77	388 ± 17
6	42.61 ± 0.90	2.59 ± 0.78	391 ± 16
7	42.34 ± 0.69	2.67 ± 0.99	401 ± 15
8	41.88 ± 0.79	2.87 ± 0.45	403 ± 14
9	41.30 ± 1.37	3.09 ± 0.69	401 ± 14
10	41.25 ± 0.78	3.09 ± 0.62	404 ± 14
11	40.77 ± 0.91	3.18 ± 0.63	404 ± 11
12	40.66 ± 1.05	3.13 ± 0.60	404 ± 12

^*^D = Diopter.

^**^CCT = central corneal thickness.

**Figure 3:**
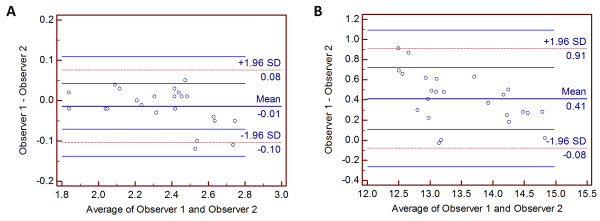
**Bland-Altman plots for observer 1 and observer 2 comparing measurements of anterior chamber depth (A) and anterior chamber width (B).** The horizontal lines represent the mean and 95% limit of agreement (LoA).

The mean values (± standard deviation) of the ACD and ACW measured by 2 independent observers are tabulated in Table 2. There was no statistical significance in the between-observer AS-OCT measurements. There was a very strong correlation between-observer for all measurements and the correlation was statistically significant (*p* < 0.001). In addition, there was a high agreement between 2 observers and the ranges of 95% LoA were acceptable from a clinical perspective (Figure 3). Observer 1 appeared to underestimate the mean ACD measurement made by observer 2, but the bias was not systematic as the difference values were scattered evenly above and below the mean bias (Figure 3A). In ACW measurement, observer 1 overestimated the mean value measured by observer 2, but the bias didn’t appear to be systematic (Figure 3B). Spans of the 95% LoA for mean ACD and ACW were 0.038 mm and 0.212 mm, respectively.

**Table 2:** Inter-observer mean anterior chamber depth and anterior chamber width of rabbit eyes measured by Visante AS-OCT

**Parameters**	**Observer 1**	**Observer 2**	***p*value**	***r*value**		**Bias (95% CI)**	
					Mean LoA^*^	**Upper limit**	**Lower limit**
Mean ACD^†^ (mm)	2.34 ± 0.24	2.36 ± 0.26	0.848	0.987	−0.014 (−0.033 to 0.005)	0.076 (0.042 to 0.109)	−0.104 (−0.138 to −0.070)
Mean ACW^‡^ (mm)	13.70 ± 0.71	13.29 ± 0.84	0.073	0.962	0.414 (0.308 to 0.520)	0.907 (0.723 to 1.09)	−0.079 (−0.263 to 0.105)

^*^Based on observer 1 - observer 2 values.

^†^ACD = anterior chamber depth.

^‡^ACW = anterior chamber width.

CI = confidence interval; LoA = limits of agreement.

**Figure 4:**
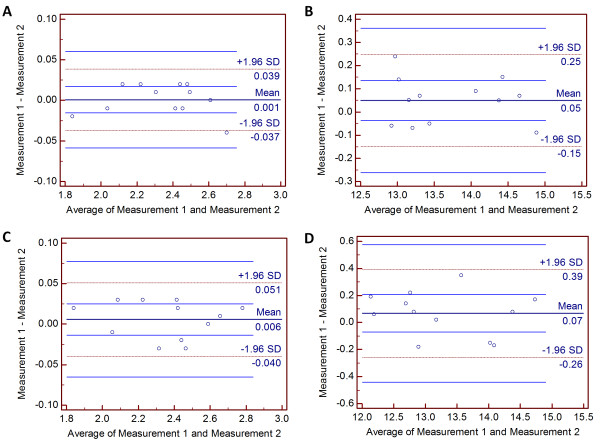
**Bland-Altman plots showing intra-observer agreement on measurements of anterior chamber depth (ACD) and anterior chamber width (ACW).** (**A**) Repeated ACD measurements made by observer 1. (**B**) Repeated ACW measurements made by observer 1. (**C**) Repeated ACD measurements made by observer 2. (**D**) Repeated ACW measurements made by observer 2. The horizontal lines represent the mean and 95% limit of agreement (LoA).

The mean values (± standard deviation) of 2 repeated measurements of the ACD and ACW performed by 2 observers are tabulated in Table 3. There was no statistical significance in the intra-observer AS-OCT measurements. There was a very strong correlation for all intra-observer measurements and the correlation was statistically significant (*p* < 0.001). In addition, there was a high agreement between 2 repeated measurements performed by both observers (Figure 4). Spans of the 95% LoA for mean ACD and ACW measured by observer 1 were 0.024 mm and 0.128 mm, respectively. Spans of the 95% LoA for mean ACD and ACW measured by observer 2 were 0.029 mm and 0.211 mm, respectively.

**Table 3:** Intra-observer variability of anterior chamber depth and anterior chamber width of rabbit eyes measured by Visante AS-OCT

**Parameters**	**Measurement 1**	**Measurement 2**	***p*value**	***r*value**		**Bias (95% CI)**	
					Mean LoA^*^	**Upper limit**	**Lower limit**
Mean ACD^†^ (mm)							
Observer 1	2.34 ± 0.25	2.34 ± 0.25	0.993	0.997	0.001 (−0.011 to 0.013)	0.039 (0.017 to 0.060)	−0.037 (−0.058 to −0.015)
Observer 2	2.36 ± 0.27	2.35 ± 0.27	0.958	0.990	0.006 (−0.009 to 0.020)	0.051 (0.025 to 0.077)	−0.039 (−0.065 to −0.014)
Mean ACW^‡^ (mm)							
Observer 1	13.73 ± 0.72	13.68 ± 0.73	0.870	0.996	0.049 (−0.015 to 0.113)	0.248 (0.134 to 0.361)	−0.149 (−0.262 to −0.036)
Observer 2	13.32 ± 0.85	13.25 ± 0.88	0.850	0.982	0.067 (−0.038 to 0.173)	0.392 (0.207 to 0.577)	−0.257 (−0.442 to −0.072)

^*^Based on measurement 1 - measurement 2 values.

^†^ACD = anterior chamber depth.

^‡^ACW = anterior chamber width.

CI = confidence interval; LoA = limits of agreement.

## Discussion

All organs go through progressive changes during growth and aging and the eye is no exception [22]. Many studies have elucidated the age-related changes in human ocular structures, such as the corneal curvature, endothelial cell density, refractive state, ACD and CCT [23-25]. However, there are only a limited number of similar studies that have been performed in animals, especially rabbits. In this study, we have shown that the CCT, corneal curvature and refractive state in the rabbits underwent a rapid change during the first year of life. We found a marked decrease in keratometry with age, and an increase in CCT with age. We also saw an increase in the spherical equivalent with age, albeit the changes were slow and gradual. In addition, also for the first time, the inter- and intra-observer reproducibility of measurements of ACD and ACW in an animal model was assessed. We demonstrated that there was a good agreement for the measurements made by two independent and masked observers.

In humans, the changes in ocular structures occur progressively over years, with greater changes occurring during adolescence. From our findings, similar changes are observed in the rabbits, albeit at a more rapid rate. For example, Asbell et al. reported a mean corneal curvature of 46.82 ± 2.11 D (range: 44.08-49.50 D) in the human newborn to 6-month old group [26]. The keratometry progressively decreased and seemed to stabilize at about 54 months. In rabbits of 1–6 month-old group, the mean corneal curvature was 45.60 ± 2.77 D (range: 51.88-41.25 D) and appeared to stabilize at about 9 months (41.30 ± 1.37 D).

Refractive development is a complicated process that can be mediated by both genetic and environmental components. In most mammalian and avian species, early development normally starts with hyperopia and the refractive error gradually decreases to emmetropization [27]. An exception was found in the mouse where the refractive development begins with hyperopic refractions, but they continues to age with increasing hyperopic refractive errors [28,29]. In this study, we found that some rabbits develop hyperopia as early as 1 month old and all rabbits age with a gradual increase in spherical equivalent refraction.

The age-related alterations in rabbit CCT were evaluated by using a RTVue Fourier-domain OCT. The fast scan speed of RTVue can overcome the effects of eye movements during measurements, leading to the possibility of higher image resolution [30]. We saw a rapid increase in the rabbit CCT for the first 7 months. The increase in the corneal thickness appeared to be stabilized after 8 months. In humans, thicker median CCT was reported at each year of age starting from 1 to 11 years old and reaching a plateau after age 11 [31].

Changes in the rabbit are, obviously, not applicable to man, for the young rabbit is generally more mature than the human infant. Because of the faster growth rate, young rabbits also elicit a more robust post-operative inflammatory response [32]. Ideally, a more mature and stable cornea should be used for experimental research in order to eliminate as many fluctuating variables as possible. Literature search reveals that many experimental corneal studies have been performed on tissue obtained from young rabbits aged 2–4 months (weight of between 2–2.5 kg) [7,10,33,34]. Herse has suggested that the routine use of corneas obtained from 3 month-old rabbits are not appropriate because of the rapid growth at this age [35]. These studies offered no clear indication at which time point the cornea is considered mature and stable. Here, we have demonstrated that the corneal keratometry, refractive state and CCT reached the growth plateau and stabilized after 8 months. Based on the past studies and our findings, there is a need for an increased awareness when using young rabbits for corneal research and especially when the age-related changes in certain ocular parameters may alter the end results of an experiment.

In this study, we have for the first time demonstrated an intra- and inter-observer reproducibility of AS-OCT measurements (ACD and ACW) in the rabbit model. Although RTVue AS-OCT produces higher resolution images than Visante AS-OCT, it can only capture a narrower portion of the anterior chamber. Therefore, we used the Visante AS-OCT for measurement of ACD and ACW as the instrument is able to produce a wider and deeper field of cross-sectional view of the anterior chamber [36]. We have shown that there was a very strong correlation (|*r*| > 0.8) in the ACD and ACW measurements made by 2 independent observers, as well as the 2 repeated measurements of ACD and ACW made by 2 independent observers. In addition, Bland-Altman plots showed agreement in rabbit ACD and ACW measurements inter- and intra-observer. There were no consistent deviations between the 2 observers and repeated measurements. The span of the 95% LoA for mean ACD measured by 2 observers was 0.038 mm, which is comparable to that reported for normal human ACD (0.035 mm) [37]. The span of the 95% LoA for mean ACD measured by observer 1 and observer 2 was 0.024 mm and 0.029, respectively. These spans of 95% LoA are relatively smaller than that reported in human (0.062 mm) [37]. To the best of our knowledge, there has been no inter-observer reproducibility study on ACW measurements using Visante AS-OCT system in human patients. Our findings show that multiple trained animal technicians may do repeated measurements on rabbits and their AS-OCT measurements are reproducible. However, extensive training is necessary for technicians with no previous experience in operating AS-OCT in order to maintain the reproducibility, as the intra-observer variation can become significant in measurements made by non-experts [38].

The mean LoA of ACW measurement performed by 2 independent observers was 0.414 mm (95% CI: 0.308 to 0.520). The greater than mean bias of ACD between observers is as expected, because of the difficult manual identification of the scleral spur as a measurement reference point for ACW. The difficulty of this practice has been well documented [39,40]. In a cross-sectional study of 502 eyes, Sakata et al. found the detectability of the scleral spur on AS-OCT images to be 72% overall [41].

One limitation of this study is the small number of rabbits used to draw the conclusion, although our findings were highly reproducible and consistent within these rabbits. Increasing the sample size would most likely reduce the *p* value of our already statistically significant results and therefore, might not change the current conclusion of the study.

## Conclusion

The CCT, corneal curvature and refractive state of the rabbits undergo a dramatic change in the first few months of life. We found that keratometry decreases with age, while CCT and spherical equivalent refraction increases with age. In corneal refractive experiments that use rabbits as an animal model, it is critical to take these ocular parametric changes into account, particularly when using younger less mature animals, as they may skew the results of the experimental model. In this study, we have also demonstrated a good intra- and inter-observer reproducibility of measurements of ocular parameters by using an AS-OCT; hence, advocating its use in pre-and post-operative assessment of the anterior segment of the eye in a rabbit experimental model.

## Abbreviations

CCT: Central Corneal Thickness; ACW: Anterior Chamber Width; ACD: Anterior Chamber Depth; AS-OCT: Anterior Segment-optical Coherence Tomography; LASIK: Laser in-situ Keratomileusis; ReLEx: Refractive Lenticule Extraction; PRK: Photorefractive Keratectomy; LoA: Limit of Agreement.

## Authors' contribution

RIA and JSM conceived and designed the experiments. AKR and NYT performed the experiments. AKR, NYT, HMH, RIA, SSC and JSM analysed the data and performed the statistical analysis. HMH, SSC and JSM contributed materials and analysis tools. AKR wrote the paper. All authors read and approved the final manuscript.
